# The Human Teeth

**Published:** 1854-09

**Authors:** 


### THE HUMAN TEETH.

I have to record here an example of the success of genius, added to that indomitable perseverance which genius only can command, in reference to the subject which heads this article. I do it the more readily, as in one of the first numbers of the Journal an intimation was given that an occasional page would be devoted to the preservation of the Teeth, as an important means not only of preserving the health, but of maintaining personal beauty. Has the reader ever seen Queen Victoria, and a —— squirrel? What would she not have given to have had a set of teeth less like the front ones of the lively little animal named?

Some long years ago, I knew Dr. A—— to be laboring after the *ne plus ultra* of dentistry; but week after week, month after month, year after year, he labored on in his little workshop, where the white heat of his furnace seemed almost sufficient to burn his eyes out or blind them with its glare: and, whether in December or July, there was the same toil,—the same cheerful hopefulness, if not actual confidence of success, as his motto seemed to be, that "what ought to be done, could be done," and that he was going to do it. Since I knew him to be thus engaged, he has grown bald, and age and wrinkles have come, but they have brought with them an enduring triumph. But, after all, what is it? He has found a silicious compound, of an exactly equal linear expansion and contraction, under the application of heat, to that of the metal upon which it is fused. Now, this is worse than Greek to the unscientific reader; but if he should live to the age of sixty years, or more, and does not care to look as old by a score or two, then this discovery will be to him a truth whose value in agreeableness and satisfaction cannot be accurately computed. The result of this discovery and invention, for it is both, is simply this: that false teeth can be made, including gums, more beautiful—because more regular—than the natural ones, more durable, more untarnishable, and more indestructible than they. These teeth can be used with comfort in eating; the acids of the mouth cannot corrode them, while the teeth themselves are so strongly clasped by the artificial gum, that it is altogether impracticable for a particle of food or the most penetrating liquid to get between them; hence, the mouth and the breath can be kept sweeter and cleaner than if the teeth were all natural. This close-fitting has never been accomplished before, because of the different expansibility of the material of which the teeth were made, and that into which the teeth were fitted,—as also of the artificial gum which was used to bind them together. In fact, a set of artificial teeth, with gum, is made, which for beauty, endurance, cleanliness, distinct articulation, comfortableness in mastication, expression, length, form and shade, has never been equalled in this or any foreign country I have ever visited. As a matter of personal convenience, agreeableness, and satisfaction to those who wear them, the discovery is literally invaluable; and if the inventor could only be induced to lay aside the diffidence which is inseparable from true talent, a career of successfulness would open before him which would satisfy his largest desire. The whole Dental world, if they could see its full truth, will be delighted to know that—

*Dr. John Allen, of Bond-street, New York, has made a discovery by which Artificial Dentures can be so constructed, that in point of strength, cleanliness, life-like appearance, and adaptation, a degree of perfection has been attained never hitherto equalled. None of the ingredients employed admit of being tarnished or corroded in the mouth, while the fusing substance, capable of any desired tint of artificial coloring, renders the whole as firm as a solid bone: and, when necessary, can be so formed as to restore sunken cheeks to their natural rotundity, and can be worn without appreciable discomfort, being kept in place wholly by atmospheric pressure.*

